# Protection of Thiol Groups on the Surface of Magnetic Adsorbents and Their Application for Wastewater Treatment

**DOI:** 10.1038/s41598-018-26767-w

**Published:** 2018-06-05

**Authors:** Inna V. Melnyk, Roman P. Pogorilyi, Yuriy L. Zub, Miroslava Vaclavikova, Karolina Gdula, Andrzej Dąbrowski, Gulaim A. Seisenbaeva, Vadim G. Kessler

**Affiliations:** 1 Chuiko Institute of Surface Chemistry NASU, Department of Surface Chemistry and Hybrid Materials, Kyiv, 03164 Ukraine; 2 Institute of Geotechnics SAS, Department of Physical and Physico-chemical Methods of Mineral Processing, Kosice, 04001 Slovak Republic; 3 Bohdan Dobrzański Institute of Agrophysics PAS, Department of Microstructure and Mechanics of Biomaterials, Lublin, 20-290 Poland; 4 Maria Curie-Skłodowska University, Faculty of Chemistry, Lublin, 20-031 Poland; 5 Swedish University of Agricultural Sciences, Department of Chemistry and Biotechnology, BioCenter, Uppsala, 750 07 Sweden

## Abstract

The magnetite nanoparticles were functionalized with silica shells bearing mercaptopropyl (monofunctional) and mercaptopropyl-and-alkyl groups (bifunctional) by single-step sol-gel technique. The influence of synthetic conditions leading to increased amounts of active functional groups on the surface and improved capacity in the uptake of Ag(I), Cd(II), Hg(II), and Pb(II) cations was revealed. The physicochemical properties of obtained magnetic nanocomposites were investigated by FTIR, Raman, XRD, TEM, SEM, low-temperature nitrogen ad-/desorption measurements, TGA, and chemical microanalysis highlighting the efficiency of functionalization and mechanisms of the preparation procedures. The removal of the main group of heavy metal cations was studied in dependence from the pH, contact time and equilibrium concentration to analyze the complexes composition for the large scale production of improved adsorbents. It was demonstrated that introduction of the alkyl groups into the surface layer prevents the formation of the disulfide bonds between adjacent thiol groups. The obtained adsorbents were employed to treat real wastewater from Ruskov, Slovakia with concentration of Fe 319 ng/cm^3^, Cu 23.7 ng/cm^3^, Zn 36 ng/cm^3^, Mn 503 ng/cm^3^, Al 21 ng/cm^3^, As 34 ng/cm^3^, Pb 5.8 ng/cm^3^, Ni 35 ng/cm^3^, Co 4.2 ng/cm^3^, Cr 9.4 ng/cm^3^, Sb 6 ng/cm^3^, Cd 5 ng/cm^3^. These materials proved to be highly effective in the removal of 50% of all metal ions, espeсially Zn, Cd, and Pb ions from it and turned recyclable, opening for their sustainable use in water purification.

## Introduction

Heavy metals are a group of elements (with a molecular mass above 50) that actively participate in biological processes forming part of many enzymes, but they have a harmful effect on the body causing poisoning and mutations at a certain concentration. These include: lead, zinc, cadmium, mercury, molybdenum, chromium, manganese, nickel, tin, cobalt, titanium, copper, vanadium. For example, сhromium (in its hexavalent form) and arsenic are carcinogens; cadmium causes a degenerative bone disease; and mercury and lead damage the central nervous system. Other heavy metals noted for their potentially hazardous nature, usually as toxic environmental pollutants, include manganese (central nervous system damage); cobalt and nickel (carcinogens); copper, zinc, selenium and silver (endocrine disruption, congenital disorders, or general toxic effects in fish, plants, birds, or other aquatic organisms).

Sources of heavy metals are divided into natural (weathering of rocks and minerals, erosion processes, volcanic activity) and man-made (mining and processing of minerals, fuel combustion, traffic, agricultural activities). There are many methods of water treatment, including mechanical, biological, physical and chemical, but adsorption is very important for removing microquantities of metal ions that remain after previous purifications. For this purpose, surface of inorganic adsorbents are modified by various complex and chelate groups^1–5^ that are capable of forming stable complexes with metal ion-pollutants. Also the advantages of inorganic sorbents are resistance to heat, radiation, organic solvents, and their high selectivity and regeneration^4–6^.

Magnetically functionalized materials (adsorbents) could be easily and quickly removed from the sorption process, and could also be easily modified by the required functional layers, depending on the task. To impart specific properties, the magnetite nanoparticles were functionalized by silanes^7–11^, hydroxyapatite^12^, polymers^13^, humic acid^14^, or can be used to produce composites with activated carbon^15^, MOF^16^ and others^17,18^.

**Figure 1:**
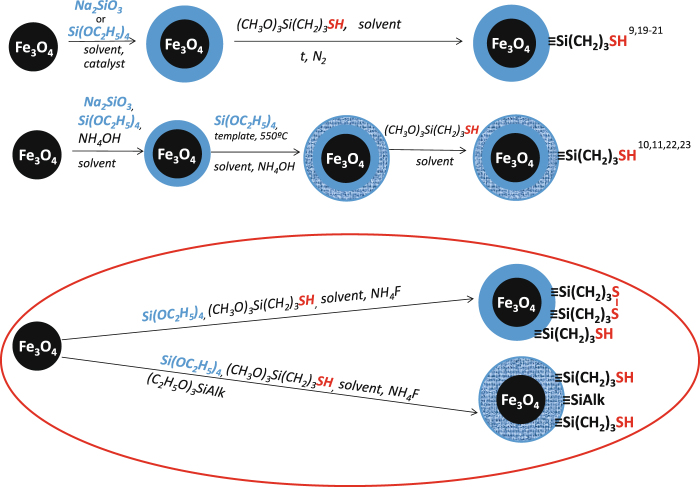
Synthesis schemes of functionalized magnetite nanoparticles with thiol groups.

It is known that such elements as Ag, Hg, Cd, Pb have a strong affinity for sulfur. Thus, the magnetite surfaces are functionalized by sulfur-containing groups for improved adsorption of above-mentioned ions from aqueous solutions. For example, silica-coated magnetic nanoparticles modified with thiol groups using 3-mercaptopropyltrimethoxysilane (MPTMS)^9–11,19–24^ have been studied (Fig. 1). Generally magnetic particles are covered with a polysiloxane layer with subsequent modification using MPTMS^9,19–21^. In some cases, surfactants have been used to form a porous surface layer^10,11,22,23^. It can be found in the literature that the as-synthesized materials were used successfully to adsorb the lead^9–11^, gold^19^, cadmium^9^, and mercury^9–11,20,21^ ions. The multistage techniques proposed in the literature result generally in materials possessing low concentration of thiol groups. This urges a requirement for application of single-step methods in preparation of magnetite particles functionalized by 3-mercaptopropyl groups^24^. Moreover, it is known that thiol groups are easily oxidized to form disulfide bonds^25,26^ that are no longer capable of ion-exchange interactions, which decrease their sorption capacity towards many metal ions^27^. Recently it has been demonstrated that introduction of an additional alkyl function into the ligand layer is capable to limit the unwanted interactions and stabilize single functional groups^28^. Therefore it appeared plausible that expanding the range of application for such particles could be achieved by creating mono- as well as bifunctional layers, containing thiol, methyl and/or propyl groups on their surface.

The novelty of this work is the study the one-pot synthesis of magnetic materials with different surface concentrations of 3-mercaptopropyl groups. This approach opens up the possibilieties for larger scale production of improved adsorbent materials and permits adjusting particle size, polysiloxane layer thickness, its porosity, and the amount of free thiol groups – the characteristics crucial for the adsorbent applications. These adsorbents were employed in the wastewater purification to study their sorption efficiency.

## Results and Discussion

### Synthesis and content of Thiol and Alkyl groups

Procedures, where the sol created by TEOS (tetraethyl orthosilicate) and MPTMS (and MTES (methyltriethoxysilane) or PTES (*n*-propyltriethoxysilane)) was added dropwise to the suspension of magnetite during 20 minutes were applied. In order to form porous silica shell over magnetite praticles, the ammonium fluoride was emloyed as catalyst^29^ in the synthesis of samples **S1**, **SM1** and **SP1**. The rest of saples **S2**, **SM2**, **SP2** and **S3** were synthetised without catalyst to compare physical charcteristics of obtained materials. The composition of the reaction mixture and content of functional groups present on the magnetic particles functionalized by 3-mercaptopropyl groups are given in Table 1. The molar ratio between Fe_3_O_4_ and TEOS (1:20), which provided stability of obtained materials in an acidic environment, was the same as used previously^30^.

**Table 1:** The composition of the reaction mixture and content of functional groups present on the magnetic particles functionalized by 3-mercaptopropyl groups.

Sample	Molar ratio of reaction components				S, % (el.anal./EDXS)	C_SH_, mmol/g		C, % (el.anal.)	C_alkyl gr._, mmol/g
	Fe_3_O_4_	TEOS	MPTMS	Alkyl		el.an.	EDXS		
**S1**	0.15	3	1	—	2.6/4.0	0.8	1.25	2.9	—
**SM1**	0.15	3	1	0.5	5.7/3.6	1.8	1.13	9.4	2.5
**SP1**	0.15	3	1	0.5	3.8/5.5	1.2	1.7	9.3	2.7
**S2**	0.15	3	1	—	0.7/0.8	0.2	0.25	0.9	—
**S3**	0.15	3	1	—	0.9/1.05	0.3	0.33	1.1	—
**SM2**	0.15	3	0.5	0.5	1.0/1.1	0.3	0.34	1.5	0.3
**SP2**	0.15	3	0.5	0.5	0.8/0.3	0.25	0.1	1.4	0.1

Table 1 shows the content of introduced functional groups calculated from the data provided by different methods. The amount of 3-mercaptopropyl groups was calculated from the elemental analysis and EDXS data. It can be seen that EDXS data correlate within an error, thus the data of elemental analysis for sulfur were used in all subsequent calculations. High concentration of 3-mercaptopropyl groups in bifunctional materials may be the result of increased rate of hydrolysis and condensation of silanes having an alkyl group in the presence of MPTMS. It was observed that the hydrolysis rate decreases with increasing length of alkyl groups connected to the silicon atom^31^. The same effect is observed for samples with 3-aminopropyl groups with simultaneous introduction of a methyl or *n*-propyl groups (bifunctional materials). Almost double increase in amount of 3-aminopropyl groups when compared with the theoretically calculated amounts can be observed as well^32^.

The amount of methyl and *n*-propyl groups in the obtained samples were evaluated based on elemental analysis (Table 1) and thermograms (Fig. FS1), where the weight loss of the part which corresponds to 3-mercaptopropyl groups (elemental analysis data for sulfur) was substracted from the total weight loss assuming that the decomposition of organic groups begins over 200 °C. Thus, the quantities of methyl (M) or *n*-propyl (P) groups were found to be 2.3 and 0.6 mmol/g in samples **SM1** and **SM2**, respectively, and 2.3 and 0.3 mmol/g in samples **SP1** and **SP2**, respectively. In addition, the amounts of alkyl groups were calculated from the elemental analysis of carbon (Table 1). It is obvious that the concentration obtained from these two methods correlate well. Smaller amount of both 3-mercaptopropyl and alkyl groups in the **S2**, **SM2** and **SP2** samples, compared to the **S1**, **SM1** and **SP1** samples, justified the importance of ammonium fluoride as catalyst. It can be explained that in the syntheses of the **S1**, **SM1** and **SP1** samples the hydrolysis and condensation of silanes proceeded to a major degree, consequently leading to formation of bigger amounts of functional groups on the material’s surface.

### IR and Raman spectra

**Figure 2:**
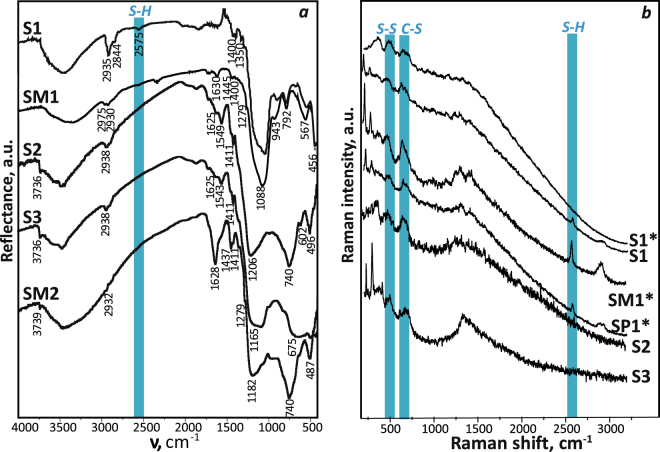
(**a**) DRIFT spectra of the samples **S1**, **SM1**, **S2**, **S3** (at 100 °C) and **SM2** (20 °C); (**b**) Raman spectra of some samples (* - samples after 2 years).

The presence of both polysiloxane layer and the functional groups, on the surface of magnetite particles, was confirmed by IR spectroscopy (Fig. 2a). Strong absorption band in the 1000–1200 cm^−1^ region is observed for all samples. This can be assigned to ν_as_(≡Si–O–Si≡) of a three-dimensional skeleton siloxane bond.

It is known that formation of the Si–O(Me) bonds on the silica surface is characterized by the appearance of absorption bands in the 930–980 cm^−1^ region^33^. The absorption band at 943 cm^−1^, which can be attributed to the Si–O(Fe) vibrations, is observed after removal of physically adsorbed water at 100 °C. The absorption band at ~600 cm^−1^ is assigned to ν(Fe-O) stretching vibrations of magnetite. It can be seen, that in the spectra of all materials, δ(Н_2_О) vibrations at 1625–1630 cm^−1^ is present, which proves the adsorbed water on the materials surface.

Groups of bands in the DRIFT spectra indicated that functional groups were also present. The presence of propyl chains was obvious from a set of bands at 1350, 1411 and 2840–2980 cm^−1^. These bands are assigned to ω(CH_2_), δ(Si-CH_2_) and ν_s,аs_(СН), respectively. In the DRIFT spectra of samples **SM1** and **SM2** the two low-intensity absorption bands at 1280 and 1437 cm^−1^, assigned to δ_s_(CH_3_) and δ_аs_(CH_3_), respectively, were present. These bands verified the presence of methyl groups in the samples. A low-intensity absorption band at 2575 cm^−1^ was assigned to the ν(SH) stretching vibrations of 3-mercaptopropyl group. This band was absent in almost all spectra, or had a very low intensity, as well as in other reported studies dedicated to functionalization of magnetite by MPTMS. This can be attributed to relatively low concentration of functional groups present on the surface^9,10^. However, one should not exclude possibility of partial oxidation of the 3-mercapto groups to disulfide. Therefore, the Raman spectra were also recorded (Fig. 2b). In these spectra could be seen three lines at 509, 640 and 2575 cm^−1^ attributed to the stretching vibrations of S–S, C–S and S–H bonds, respectively^34,35^. It could be observed that all spectra contained lines attributed to C–S and S–S stretching vibrations. The latter confirms the presence of the disulfide bridges which apparently formed in the oxidation of 3-mercaptopropyl groups.

Only three of them, two bifunctional **SM1** and **SP1**, and freshly synthesized sample **S1**, contained the line assigned to the S–H stretching vibrations. The intensity of this line also represents the number of functional groups per unit of the surface and correlates with the data presented in the Table 1. Thus, the introduction of alkyl groups to the surface layer of magnetically sensitive materials with 3-mercaptopropyl groups partially inhibits their oxidation.

### Powder XRD

**Figure 3:**
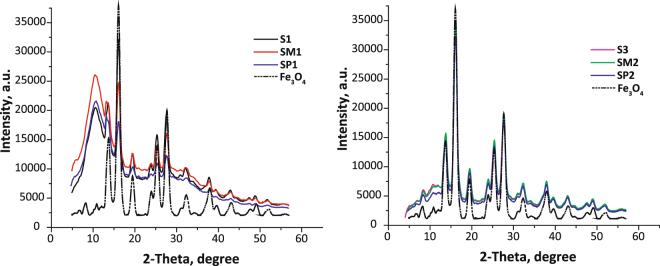
XRD patterns of the samples.

Figure 3 shows the XRD patterns of the functionalized samples. The reflections at 13.8, 16.2, 16.8, 19.5, 23.8, 25.4, 27.6, 31.0, 32.2, 32.5, 36.8, 38.8 (2-theta) correspond to the crystalline phase of magnetite Fe_3_O_4_ cubic system (JCPDS No. 19–629). Moreover, the thickness of the polysiloxane layer on the magnetite particles surface, can be seen from the diffraction patterns. Thus, an increase in polysiloxane shell for **S1**, **SM1**, **SP1** samples may indicate the presence of broadened reflex at 10.5 (2-theta), the appearance of which can be attributed to the scattering of X-rays due to the presence of a certain amount of amorphous silica^36^. This reflex has low intensity with a minimum content of the polysiloxane layer (Fig. 3 for **S2**, **SM2**, **SP2** samples) in the diffraction patterns.

The crystal size of initial magnetite and nanocomposite particles was estimated from the XRD patterns by using Debye−Scherrer’s equation^16^. The crystallite size of magnetite was found to be about ∼14.2 nm. The particle diameter is ∼16.4 nm for the samples **S2**, **S3**, **SM2**, **SP2**. However, the diameters of particles for **S1**, **SM1** and **SP1** samples calculated from this equation were 21.8 nm, 26.4 nm and 29.9 nm, respectively, indicating that a larger modifying layer was formed for the samples obtained with the catalyst.

### TEM and SEM

**Figure 4:**
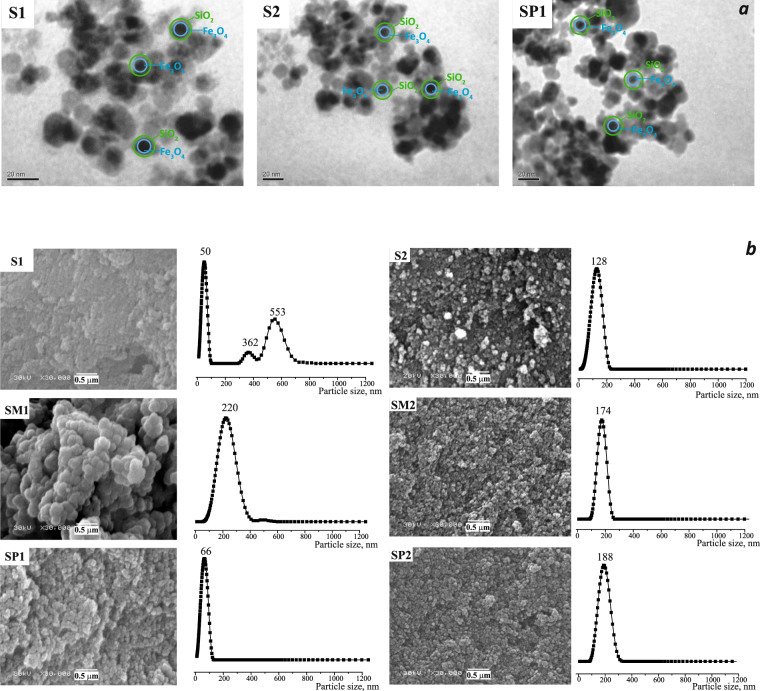
TEM images of selected samples (**a**) and SEM images and particle size distribution using photon cross-correlation spectroscopy of the samples with mono- and bifunctional surface layer (**b**).

TEM microphotographs for exemplary samples shown in the Fig. 4a confirmed that the pristine magnetite particles have a spherical shape and an average size of ∼12 nm (blue circle). The gray substrate which can be attributed to the polysiloxane part (green circle) of the composite^3,20^, can be observed. The same images can be noticed for similar magnetite/silica samples obtained by two and more stage, in which the magnetic particles are incorporated in polysiloxane shells^21^. From TEM photos the size of a magnetic particle with a shell was estimated, it was about ∼20 nm. The average particles size is in agreement with XRD data.

Figure 4b shows the SEM microphotographs of the samples. The average diameters of spheres were calculated from these data presented in the Table 2. Based on TEM we found that the size of the functionalized particles is about 20 nm, however the spherical agglomerates are seen on SEM images. Largest agglomerates were obsereved for sample **SM1**. This may result from the higher condensation rate of MTES during synthesis. The particle size is gradually increased due to increasing size of a polysiloxane shell covering magnetic cores. Comparing these data with those obtained by photon cross-correlation spectroscopy (the last column in the Table 2), it can be concluded that for the first three samples are correlated. However, differences were observed for sample **S1**, where not only single magnetic particles, but also their agglomerates are incorporated into silica shell. Regarding to samples **S2**, **S3**, **SM2** and **SP2**, the dimensions obtained from photon cross-correlation spectroscopy (PCCS) are almost two times higher than those from SEM. This may indicate a thin or insular polysiloxane shell covered magnetic particles, which not prevent their aggregation sufficiently. Similar agglomerates of functionalized particles are observed also for samples obtained in several stages^20^. However, from the practical point of view, if the particles form aggregates in air or solution, the PCCS method will reveal it. Thus, we can evaluate the real state of particles in which they are used.

**Table 2:** The size, structure and sorption characterization of the obtained magnetosensitive adsorbents.

Sample	C_SH_, mmol/g elem.an.	C_SH,_ mmol/m^2^	C_SH,_ gr./nm^2^	SC, mmol/g				S_sp._, m^2^/g	d, nm from SEM	d, nm from PCCS
				Ag^+^	Hg^2+^	Cd^2+^	Pb^2+^			
**S1**	0.8	0.024	14	0.16	0.8	0.63	0.4	33	70	50, 362
**SM1**	1.8	0.005	3	0.68	1.78	1.33	0.8	360	280	220
**SP1**	1.2	0.004	2	0.46	1.19	0.82	0.44	290	60	66
**S2**	0.2	0.002	1	—	—	—	—	125	80	128
**S3**	0.3	0.004	2	0.26	0.3	—	—	80	80	181
**SM2**	0.3	0.003	2	0.18	0.2	—	—	107	90	174
**SP2**	0.25	0.002	1	—	—	—	—	109	100	188

### Low-temperature nitrogen adsorption/desorption isotherms

**Figure 5:**
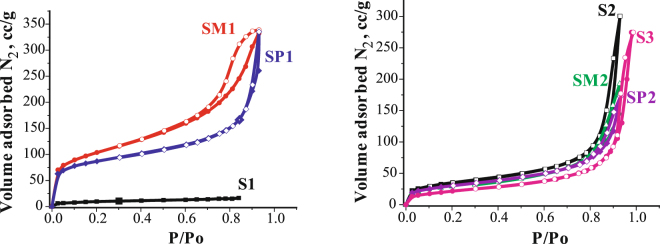
N_2_ adsorption (•) – desorption (◦) isotherms of the samples.

of the samples are shown in the Fig. 5. Values of the specific surface areas calculated from isotherms are shown in the Table 2. It is noteworthy that the isotherms determined for each sample can be attributed to different types according IUPAC classification. Isotherms of bifunctional materials synthesized in the presence of catalyst (**SM1** and **SP1** samples), can be attributed to type IV; sample **S1 -** type I; the rest of the samples - type II. The sample **S1** has the lowest surface area, which can be caused by the presence of agglomerates 360 nm and more in diameter. In the type IV of isotherm (bifunctional samples **SM1** and **SР1**) a capillary-condensation hysteresis loop (Fig. 5) can be observed. This type of isotherms is typical for xerogels^37^, and indicates the presence of mesopores in the material structure. The way of adding a sol to the magnetite suspension, as well as the presence of F^-^ catalyst^29^ during synthesis influenced the isotherm shapes and specific surface areas. Thus, for **SM1** and **SP1** samples, gradual introduction of sol to the mixture and the presence of NH_4_F, resulted in a rapid increase of the adsorbed N_2_ at the initial region of isotherm higher values of the specific surface. This indicated the formation of a porous surface layer. The values of specific surface areas for **S2**, **SM2**, **SP2** and **S3** samples, does not significantly differ from the original magnetite^38^ that can be attributed to a slight difference in the particle size of the samples.

### Sorption study

The effect of initial pH on metal ions adsorption onto **SP1** composite was studied for Pb(II) and Cd(II) solutions (Fig. FS2). The results demonstrate that the maximum of adsorption capacity appear at pH 5–6 for both metal ions. The highest sorption of lead ions at a similar pH is observed for mercapto functional groups^39^ as well as for others^40^. pH was chosen ∼2 for mercury (II) and silver (I) ions adsorption on the basis of literature data for silica materials with thiol-containing functional groups^11^.

The study of the adsorption kinetics of silver(I), mercury(II) and lead(II) ions for magnetically removable adsorbents allowed to determine the time needed to reach the equilibrium. The kinetic curves for the adsorption of metal ions are shown in the Fig. FS3. It can be seen that the equilibrium for Hg(II) and Ag(I) is reached in about 3 hours, and in about 6 hours for Pb(II). Thus, the above-mentioned times were chosen to carry out the adsorption measurements, leading to plot the adsorption isotherms.

**Figure 6:**
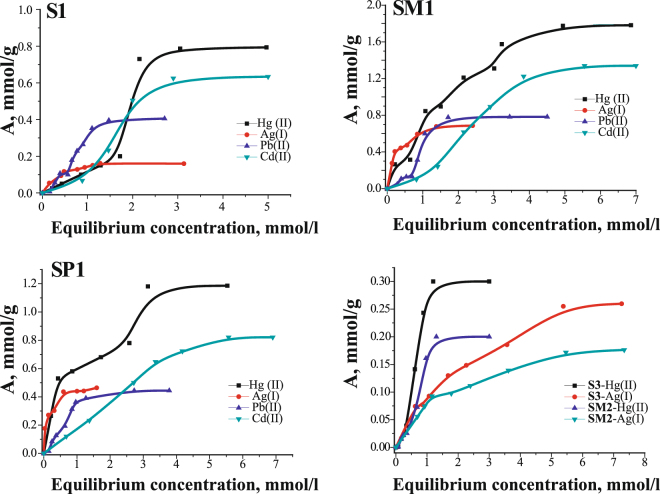
Adsorption isotherms of Ag (I), Hg (II), Cd(II) and Pb (II) ions for some magnetic thiol-containing adsorbents.

It has been known that all thiols have an affinity for mercury ions as well as sulphides^25,41^. Therefore, it is not surprising that Me/Lig ratio is almost 1/1 for all studied samples. The constants calculated from the Langmuir and Freundlich equations, are presented in the Table 3. Regarding the calculated values, the equation of Langmuir isotherm is suitable only for **SP1** sample. Ions’ adsorption on the rest of materials surfaces is better described by the Freundlich equation. Interestingly, the shape of isotherms for the silver(I) ions are similar to the Langmuir type, thus they are better described by this equation for all samples. The adsorption capacities [in mmol/g] of Ag(I) ions are lowest, when compared with those obtained for Hg(II) ions. This may be due to the formation of disulfide groups at the surface of functionalized magnetite samples during storage, with which silver(I) ions cannot interact. This may be confirmed in the case of the sample **S1** which has apparently the highest number of disulfide bridges and the lowest adsorption capacity, among **S1**, **SM1** and **SP1** samples. Furthermore, according to the analysis of the number of functional groups per 1 nm^2^, see Table 2, this sample had higher amount of functional groups onto its surface, when compared to other materials. Bifunctional materials, characterized by better developed surface, as well as the presence of the methyl or *n*-propyl groups, partially prevent the oxidation of 3-mercaptopropyl groups. Cadmium (II) ions can interact both 3-mercaptopropyl groups and disulfide bridges^42^, so Me/Lig ratio is close to 1/1. Presence of only part of available 3-mercaptopropyl groups were also confirmed by the results of adsorption of Pb(II) ions (Table 2 and Fig. 6). It can be seen that in this case Me/Lig are 1/2. Moreover, the obtained adsorption capacities of Pb(II) ions are lower that capacities obtained for Hg(II) and Ag(I) ions. In the case of the sample **SP1** containing propyl groups, it may be caused by greater distance between particular mercapto groups present on silica surface.

**Table 3:** Parameters of mercury(II), silver(I) and lead(II) adsorption obtained by Langmuir and Freundlich isotherm equations.

Sample	Me/Lig ratio	K_d_, cm^3^/g	Langmuir isotherm			Freundlich isotherm	
			a_max,_ mmol/g	*K*_*L*,_ L/mmol	*R* ^2^	*K*_*F*_	*R* ^2^
		***Ag(I)***					
**S1**	1/0.2	182.0	0.177	3.649	0.991	0.133	0.819
**SM1**	1/0.4	1642.8	0.764	4.042	0.994	0.582	0.906
**SP1**	1/0.4	849.9	0.489	9.113	0.993	0.434	0.958
**S3**	1/0.8	55.6	0.377	0.317	0.967	0.092	0.983
**SM2**	1/0.7	79.9	0.228	0.473	0.983	0.075	0.953
		***Hg(II)***					
**S1**	1/1	116.3	Not determ	Not determ	—	0.145	0.916
**SM1**	1/1	674.4	2.64	0.353	0.846	0.648	0.904
**SP1**	1/1	1232.5	1.39	0.863	0.905	0.6	0.887
**S3**	1/1	126.5	Not determ	Not determ	—	0.210	0.924
**SM2**	1/0.8	126.5	Not determ	Not determ	—	0.126	0.924
		***Cd(II)***					
**S1**	1/0.8	157.6	2.59	0.057	0.261	0.141	0.588
**SM1**	1/0.7	458.4	Not determ	Not determ	—	0.054	0.71
**SP1**	1/0.7	196.7	2.35	0.091	0.249	0.186	0.862
		***Pb(II)***					
**S1**	1/0.5	430.2	1.27	0.224	0.107	0.237	0.804
**SM1**	1/0.4	308.8	Not determ	Not determ	0.437	0.270	0.809
**SP1**	1/0.4	492.3	0.61	0.891	0.909	0.239	0.761

The distribution coefficients are presented in Table 3. Their values emphasize the high affinity of the surface layer of the bifunctional sorbents to the metal ions.

From the Table 4 it can be concluded that the synthesized samples possess high values of SC in relation of Cd(II), Hg(II), and Pb(II) ions, in comparison with the similar materials described in literature. Therefore, the methods of syntheses proposed in this paper make it possible to obtain magnetically removable adsorbents, characterized by high adsorption capacities in relation of heavy metals ions.

**Table 4:** Comparison of sorption capacities for magnetic thiol-containing nanocomposites (mmol/g).

Sorbent	Cd(II)	Hg(II)	Pb(II)	References
γ-MPTMS-SCMNPs	0.40	0.42	0.34	^9^
TMMM	0.04	0.92	0.55	^10^
SH-mSi@Fe_3_O_4_	—	1.29	0.44	^11^
Fe_3_O_4_@SiO_2_-SH	—	0.66	—	^20^
Fe_3_O_4_@SiO_2_-SH	—	0.74	—	^21^
TF-SCMNPs	—	1.04	—	^22^
Magnetic mesoporous nanocomposite particles	1.33	1.783	0.83	^23^
HBS-SH	0.35	—	0.63	^39^
Nano-adsorbent	—	—	0.82	^40^
SH-Fe_3_O_4_-NMPs-1	—	1.28	—	^44^
CNM	—	0.82	—	^45^
**S1**	0.63	0.8	0.4	This work
**SM1**	1.33	1.78	0.8	This work
**SP1**	0.82	1.19	0.44	This work
Sorbent - DMSA-Fe_3_O_4_	—	1.13	—	^46^

**Figure 7:**
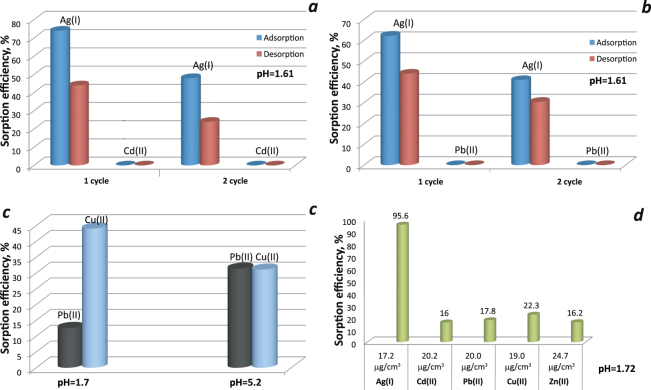
The removal efficiency of heavy metals for sample **SP1** from two-component (**a**–**c**) and five-component metal ions mixture.

The selective adsorption of **SP1** to capture other heavy metal ions in the presence of Ag^+^ was studied using binary solutions containing equal concentrations of Ag^+^ and Pb^2+^ (or Cd^2+^) at pH = 1.61 (Fig. 7a,b). The sorption was carried out from 1.2 M solutions due to this sample has 1.2 mmol/g thiol groups. We can summarize that this material showed very high affinity to Ag (I) in this condition. This is not surprising, since at such pH, as shown by our studies (Fig. FS2) and other authors^40^, the sorption of lead(II) and cadmium(II) ions is very low. Thus when all the reaction sites are occupied by silver(I) ions, the sorption of other metals does not occur. However, desorption and re-adsorption under such conditions decreases in the second cycle of using the adsorbent by about 30%, which is due to the poor solubility of silver sulphide.

We also observe that lead(II) adsorption is low in acid medium (about 12%), but about 44% of copper(II) is sorbed during sorption of divalent metals from mixtures with different pH and equal concentration (1.2 M). In a neutral medium, the sorption capacity of both metal ions is the same (Fig. 7c). The sorption for these metals increases with increasing pH^14^.

However, if sorption is carried out in acidic medium and the concentration of metals is reduced to microquantities, then silver is sorbed about 95%, and all other metals up to 20% (Fig. 7d). That is, based on the studies carried out, it can be concluded that the selectivity of metal ion extraction depends on the solution pH, ion concentration and the presence of free reaction sites.

### Wastewater treatment

**Figure 8:**
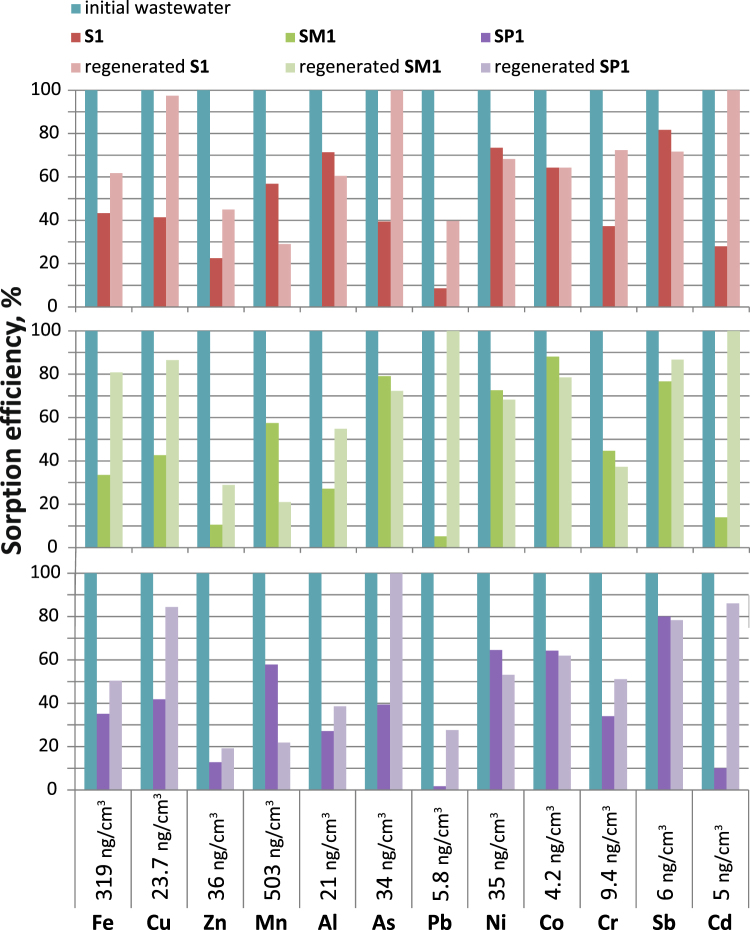
The removal efficiency of heavy metals for samples **S1**, **SM1** and **SP1** and the regenerated samples from wastewater.

Figure 8 shows the the removal efficiency of heavy metals from real wastewater in Ruskov, Slovakia. Accoding the law of Slovak republic for drink water parameters (N496 from 08.12.2010) concentration of Fe, As, Sb and Cr exceeds the permissible standards, the concentration of Cd is on the edge. Efficiancy of our materials we can estimate as we can see from Fig. 8, most metals are removed by 50%. The heavy metal ions with strong affinity to –SH such as Zn(II), Cd(II), and Pb(II), first bind with sorbtion sites, as the concentration of functional groups are large, there are many free sorption sites for other metals. Therefore, more 3-mercapto groups become accessible even to those ions with weaker affinity.

Adsorption of metal ions by recovered samples decreases, but especially for the monofunctional sample **S1** (Fig. 8). This method of regeneration was selected in order to clean the surface of the sorbent as possible organic pollutants. The partial reduction of the sorbent can be due to formation of insoluble sulfur compounds, even in HNO_3_ acid.

## Conclusions

The magnetite nanoparticles coated with polysiloxane layers containing up to 2 mmol/g of mercapto groups were synthesized in a one-step synthesis method. It was revealed that the samples with bifunctional surface layer had twice more higher content of 3-mercaptopropyl groups than monofunctional produced in the same conditions due to the different rates of the silanes’ hydrolysis. At the same time, alkyl groups can protect 3-mercaptopropyl groups from formation of disulfide bridges on the particle surface helping to maintain the functionality.

The effect of the catalyst presence in the magnetite suspension was studied. It was demonstrated that the stepwise adding of the sol and fluoride ions as catalyst contribute to increased number of thiol groups as well as better coverage of magnetite with polysiloxane layer characterized by well-developed surface. Such functional composite materials can be easily removed from the reactive medium using magnet wherethrough retain their magnetic properties. The synthesized functionalized magnetite nanoparticles can be used as effective solid phase extractants for Ag(I), Cd(II), Hg(II), and Pb (II) ions from solutions. Samples containing mercapto and alkyl groups together on the surface have shown higher removal efficiency of metal ions from real wastewater, such as up to 89% for Zinc(II), 90% for Cadmium(II), and 98.3% for Lead(II) ions. It was shown that after regeneration by nitric acid, the sample with 3-mercaptopropyl and propyl groups could be used for the second cycle of water treatment.

## Methods

### Chemicals

Following compounds were used for the syntheses: tetraethyl orthosilicate, Si(OC_2_H_5_)_4_ (TEOS, Aldrich, 98%); 3-mercaptopropyltrimethoxysilane, (CH_3_O)_3_Si(CH_2_)_3_SH (MPTMS, Aldrich, 95%); methyltriethoxysilane, (C_2_H_5_O)_3_SiCH_3_ (MTЕS, Aldrich, 99%); *n*-propyltriethoxysilane, (C_2_H_5_O)_3_Si(CH_2_)_2_CH_3_ (PTES, Fluka, 97%); iron(II) chloride, FeCl_2_·4H_2_O (Aldrich, 98%); iron(III) chloride, FeCl_3_·6H_2_O (Merck, 98%); ammonium fluoride, NH_4_F (Fluka, 98%); ammonia, NH_4_OH (Merck, 25%); ethanol, C_2_H_5_OH (Aldrich, 96%); hydrochloric acid, HCl (Aldrich, 35%). Reagents which were used in sorption process: Mercury(II) nitrate monohydrate, Hg(NO_3_)_2_·H_2_O, Silver nitrate, AgNO_3_, Lead(II) nitrate Pb(NO_3_)_2_ (reagent grade, Macrochem, Ukraine); Cadmium nitrate tetrahydrate, Cd(NO_3_)_2_·4H_2_O (ITES s.r.o.Vranov, 99%); Copper(II) nitrate trihydrate, Cu(NO_3_)_2_⋅3H_2_O (98%, ITES s.r.o. Vranov, Slovakia); Zinc(II) nitrate hexahydrate, Zn(NO_3_)_2_⋅6H_2_O (pure p.a., POCH s.a., Poland); ammonium chloride, NH_4_Cl, sodium nitrate, NaNO_3_, sodium chloride, NaCl (chemically pure, Macrochem, Ukraine); nitric acid, HNO_3_; ethylenediaminetetraacetic acid, C_10_H_16_N_2_O_8_ (EDTA); magnesium sulfate, MgSO_4_·7H_2_O - fixanal concentrates (Reahim, Ukraine); eriochrome black T (analytical grade, Reanal, Ukraine). All reagents were used as received, without further treatment.

### Syntheses

Magnetite was prepared by coprecipitation from iron(II) and iron(III) chlorides with ammonia, in nitrogen atmosphere^43^. Obtained Fe_3_O_4_ particles were spherical with average diameter about 12 nm, and specific surface area of about 96 m^2^/g. They were kept in a refrigerator in an ethanol suspension, which concentration was determined by TGA.

We began to develop methods of synthesis for samples **S1**, **SM1**, **SP1** in^24^. The samples **S2**, **S3**, **SM2**, **SP2** were prepared via similar procedure, but the distinctive feature being the addition of a sol to the magnetite suspension. This single-step procedure was carried out without catalyst.

Sample **S1** (Fe_3_O_4_/SiO_2_/-(CH_2_)_3_SH). The batch of Fe_3_O_4_ (100 mg) was dispersed in 50 cm^3^ of distilled water and treated by ultrasound for 10 min. The sol consisting of the silanes was prepared by prehydrolysis of TEOS and MPTMS (molar ratio 3/1). Then, the sol was added dropwise to magnetite suspension containing NH_4_F as catalyst (~0.5 сm^3^ was added in every 15 min) during two hours, with continuous stirring with a mechanical stirrer. After addition of the last portion, stirring was continued for another 30 min. Therefore, the total stirring time was 2.5 hours. A dark-brown sediment was separated by magnet, rinsed three times with water (50 cm^3^) and twice with ethanol (50 cm^3^). The material was then dried at ~100 °C in an oven during 24 h.

Sample **S2** (Fe_3_O_4_/SiO_2_/-(CH_2_)_3_SH). Methods of preparation the magnetite suspension and functionalized sol were similar as for sample **S1**. Unlike to the previous synthesis, the sol of silanes was added to magnetite suspension dropwise during 20 min without catalyst. Consequently, the total mixing time was 6 hours.

Sample **S3** (Fe_3_O_4_/SiO_2_/-(CH_2_)_3_SH). Method of synthesis of this sample was the same as for the sample **S2** with one exception - after prehydrolysis of TEOS, MPTMS was heated during 20 min, after that this sol was cooled and added to magnetite suspension dropwise.

Sample **SM1** (Fe_3_O_4_/SiO_2_/-(CH_2_)_3_SH/-CH_3_) was synthesized according the method described for synthesis of the sample **S1**, except that 0.49 сm^3^ of MPTMS and 0.26 сm^3^ of MTES were added to prehydrolyzed TEOS.

Sample **SM2** (Fe_3_O_4_/SiO_2_/-(CH_2_)_3_SH/-CH_3_) was synthesized according the method described the synthesis of the sample **S2**, but 0.25 сm^3^ of MPTMS and 0.26 сm^3^ of MTES were added to prehydrolyzed TEOS.

Sample **SP1** (Fe_3_O_4_/SiO_2_/-(CH_2_)_3_SH/-C_3_H_7_) was synthesized according the method described the synthesis of the sample **S1**, but 0.49 сm^3^ of MPTMS and 0.3 сm^3^ of PTES were added to prehydrolyzed TEOS.

Sample **SP2** (Fe_3_O_4_/SiO_2_/-(CH_2_)_3_SH/-C_3_H_7_) was synthesized according the method described the synthesis of the sample **S2**, but 0.25 сm^3^ of MPTMS and 0.3 сm^3^ of PTES were added to prehydrolyzed TEOS.

### Ag(I), Hg(II), Cd(II) and Pb(II) adsorption experiments

Silver(I), mercury(II), cadmium (II) and lead(II) ions adsorption from aqueous solutions of their nitrate salts was carried out with a batch adsorption process: 0.01 g of each adsorbent was contacted with 10 cm^3^ of solution of respective salt with different concentrations. All adsorption measurements were carried out at 25 °C for 16 h. Ionic strength of the solutions (0.1М) was adjusted with 1М NaNO_3_. Selective adsorption experiments were measured at the same condition using binary agueous solutions at different pH from 1.2 M solutios for 3 h. The same scheme was used for 5 metal ions mixture, the concentration of metal are in Fig. 7d. After adsorption, the adsorbents were recovered by magnet and washed with distilled water or 0.01 M HNO_3_.

For regeneration 10 cm^3^ of 0.01 M HNO_3_ solution containing 4% thiourea was added to the magnetic materials to desorb the silver.

Ag^+^, Pb^2+^, Cd^2+^, Zn^2+^ and Cu^2+^ concentrations before and after adsorption were determined using atomic absorption spectrometer С-115-М1 or with Varian AA 240 FS. The concentration of mercury(II) in the initial solutions and after adsorption process were determined by complexometric back-titration of the excessive EDTA with 0.025 М solution of MgSO_4_ (eriochrome black T as indicator, ammonium buffer solution).

The metal ions sorption equilibrium data were correlated with both, Langmuir and Freundlich models. Langmuir isotherm is described by the following equation (1):

1 $$\frac{{C}_{{\rm{eqv}}}}{{a}_{{\rm{eqv}}}}=\frac{1}{{K}_{L}}\times {a}_{{\rm{\max }}}+\frac{1}{{a}_{{\rm{\max }}}}\times {C}_{\mathrm{eqv}}$$

where *C*_eqv_ - the concentration of solute remaining in solution after equilibrium to be reached (mmol/L); *a*_eqv_ - the amount of solute adsorbed in the same condition (mmol/g); *a*_max_ - the maximum adsorption capacity in the monolayer and *K*_L_ is the equilibrium constant of the adsorption process.

The Freundlich isotherm is described by the following equation (2):

2 $$\mathrm{ln}\,q=\,\mathrm{ln}\,{K}_{F}+\frac{1}{{n}}\times \,\mathrm{ln}\,{C}_{{\rm{eqv}}}$$

where *q* - the amount of solute adsorbed (mmol/g); *C*_eqv_ - the concentration of solute remaining in solution after equilibrium (mmol/L); *K*_F_ - parameter related to maximum adsorption capacity in the multilayer of the adsorbent.

Distribution coefficients (K_d_) of the corresponding ions in case of microquantities sorption were calculated using the formula (3):

3 $${K}_{{\rm{d}}}=[\frac{{A}_{{\rm{o}}}-{A}_{{\rm{e}}}}{{A}_{{\rm{e}}}}]\times \frac{V}{{{m}}_{s}}$$

where *А*_o_ and *А*_e_ are the initial and equilibrium concentrations of the ions in solution, respectively, mmol/dm^3^; *V* is the liquid phase volume, cm^3^; and *m*_s_ is the sorbent weight, g.

### Wastewater treatment

In order to evaluate the efficacy of these adsorbents, **S1**, **SM1** and **SP1** were tested in a real wastewater treatment. Wastewater first of all was filtered through a paper filter, and after that through 0.2 microm membrane filter. Initial pH was 8.26. An adsorption set of experiments was conducted the sample dose 2.0 g/dm^3^ in 50 cm^3^ capped polyethylene vials containing 25 cm^3^ of effluent. The vials were placed inside constant orbital shaking at room temperature. The samples were collected 24 hours, filtered through paper filters, and the metal ions concentrations were quantified by Agilent Technologies 7700 Series ICP-MS.

Nitric acid 1 M was used for the regeneration of the magnetic adsorbents (1 h). After that they were dried during 3 h at 100 °C and used again.

### Characterization

The analysis for sulfur was performed at the KFK-2 equipment (Error ± 0.3 wt%). Moreover, the estimation of the amounts of the ligand was carried out by a SEM-EDS technique using a Hitachi TM-1000-µ-DeX tabletop scanning electron microscope (Error ± 0.2 wt%). The analysis for carbon of bifunctional samples was performed by elementary analyzer Vario MACRO cube (Elementar Analysensysteme GmbH, Germany) using thermal conductivity detector (Error ± 0.2 wt%).

DRIFT spectra in the range of 4000–400 cm^−1^ were recorded on a spectrometer Thermo Nicolet Nexus FTIR using diffuse reflectance “SMART Collector” at a resolution of 8 cm^−1^. Samples were mixed with KBr (for IR-spectroscopy, Aldrich) at 1:30 ratio. The data were processed by the software of spectrometer supplier company. A special thermovacuum Collector II attachment was used to register the IR spectra at higher temperatures.

The Raman spectra were recorded in the Raman microscope (inVia Reflex, Renishaw) with Raman dispersive system, using the 785 nm semiconducting laser. In order to avoid sample overheating 0.5 mW of laser power was used. The spectra of all samples were recorded at room temperature.

Thermal characterization of the prepared powders was studied using a Perkin-Elmer Pyris-1 TGA instrument connected with gas analysis unit operated with a Perkin-Elmer Spectrum 100 FTIR instrument (Error ± 0.02%).

The morphology of nanoparticles was characterized with the Analytical Scanning Electron Microscope JEOL JSM-6060 LA. In order to prevent the accumulation of surface charge and to increase the image contrast, the samples were covered with the shell of gold by cathode sputtering (JEOL Fine Coat, Ion Sputter JFC-1100). The nanoparticles were also characterized with the Transmission Electron Microscope JEOL: JEM-1230.

The granulometric analysis was carried out by using photon cross-correlation spectroscopy (PCCS) using a Nanophox particle size analyzer (Sympatec, Germany). A portion of aqueous suspension of each nanoparticles was diluted with the stabilizer to achieve a suitable concentration for measurement. The measurements were repeated three times for each sample.

X-ray powder diffraction (XRD) patterns were obtained using a Bruker SMART APEX-II diffractometer equipped with MoKα radiation (λ = 0.71073 Å). The diffraction was registered in rotation mode (1 degree/s) for powder samples sealed in 0.7 mm Lindeman tubes. Bruker APEX-II and EVA software were used for integration and data treatment.

The low temperature nitrogen adsorption-desorption isotherms were recorded with a Kelvin-1042 instrument (Costech Microanalytical) at –196 °C. The samples were pre-degassed at 110 °C for 3 hours. The specific surface areas were calculated by the BET method.

## Electronic supplementary material

Supplementary materials
