# Supplementary material for: Protection of Thiol Groups on the Surface of Magnetic Adsorbents and Their Application for Wastewater Treatment
Source: Sci Rep. 2018 Jun 5;8:8592. doi: 10.1038/s41598-018-26767-w (PMC5988753; doi:10.1038/s41598-018-26767-w)
Supplement: Supplementary file 1 — Supplementary materials [file 41598_2018_26767_MOESM1_ESM.doc]

**SUPPLEMENTARY**

**Protection of thiol groups on the surface of magnetic adsorbents and their application for wastewater treatment**

**Inna V. Melnyk^1,2*^, Roman P. Pogorilyi^1^, Yuriy L. Zub**†^1^**, Miroslava Vaclavikova^2^, Karolina Gdula^3,4^, Andrzej Dąbrowski^3^, Gulaim A. Seisenbaeva^5^, Vadim G. Kessler^5^**

^1^Chuiko Institute of Surface Chemistry NASU, Department of Surface Chemistry and Hybrid Materials, Kyiv 03164, Ukraine

^2^Institute of Geotechnics SAS, Department of Physical and Physico-chemical Methods of Mineral Processing, Kosice 04001, Slovak Republic

^3^Maria Curie-Skłodowska University, Faculty of Chemistry, Lublin 20-031, Poland

^4^Bohdan Dobrzański Institute of Agrophysics PAS, Department of Microstructure and Mechanics of Biomaterials, Lublin 20-290, Poland

^5^Swedish University of Agricultural Sciences, Department of Chemistry and Biotechnology, BioCenter, Uppsala 750 07, Sweden

*[in.melnyk@gmail.com](mailto:in.melnyk@gmail.com)

† Deceased 30 May 2016


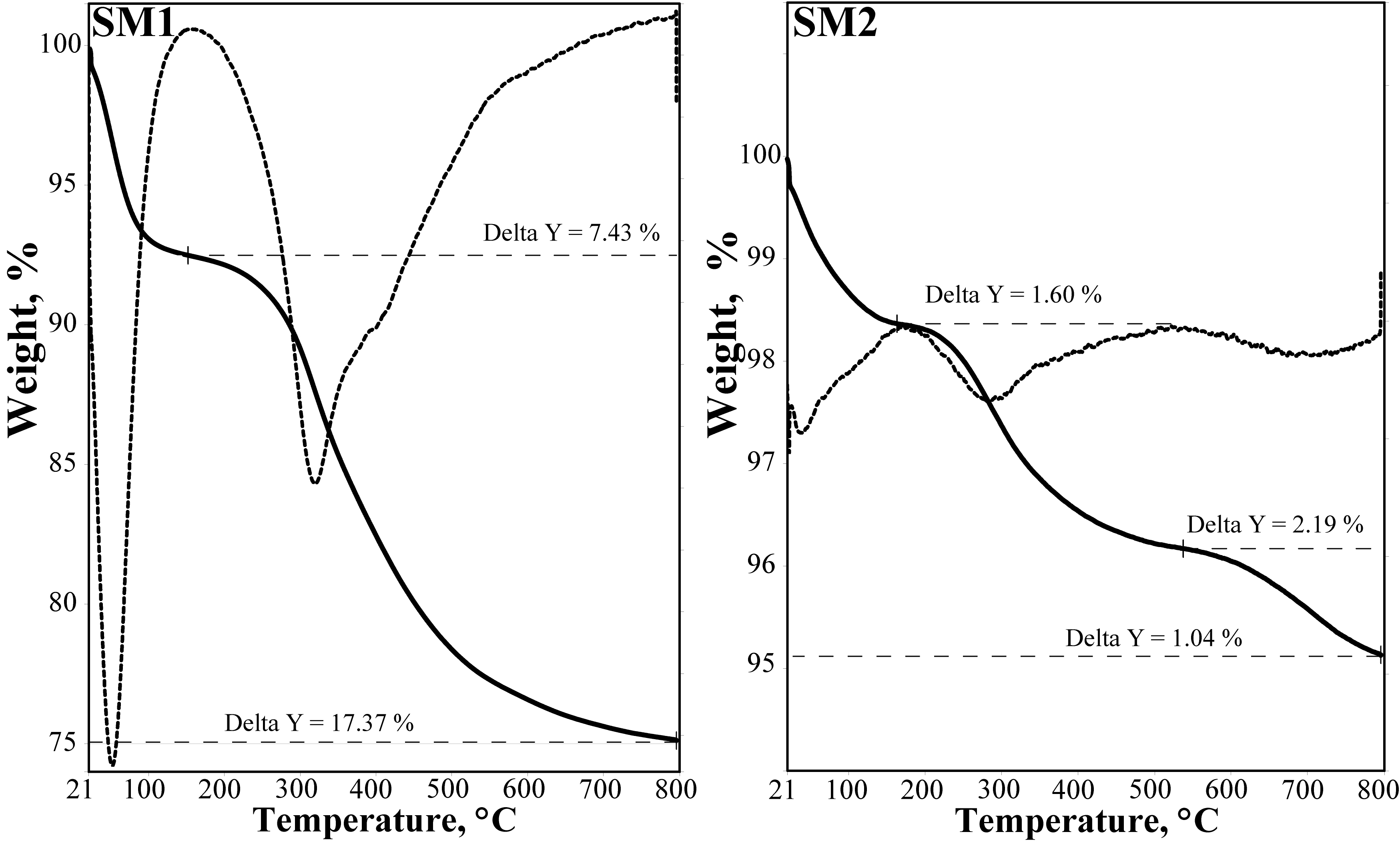


**Figure FS1.** Thermograms for samples with bifunctional surface layers.


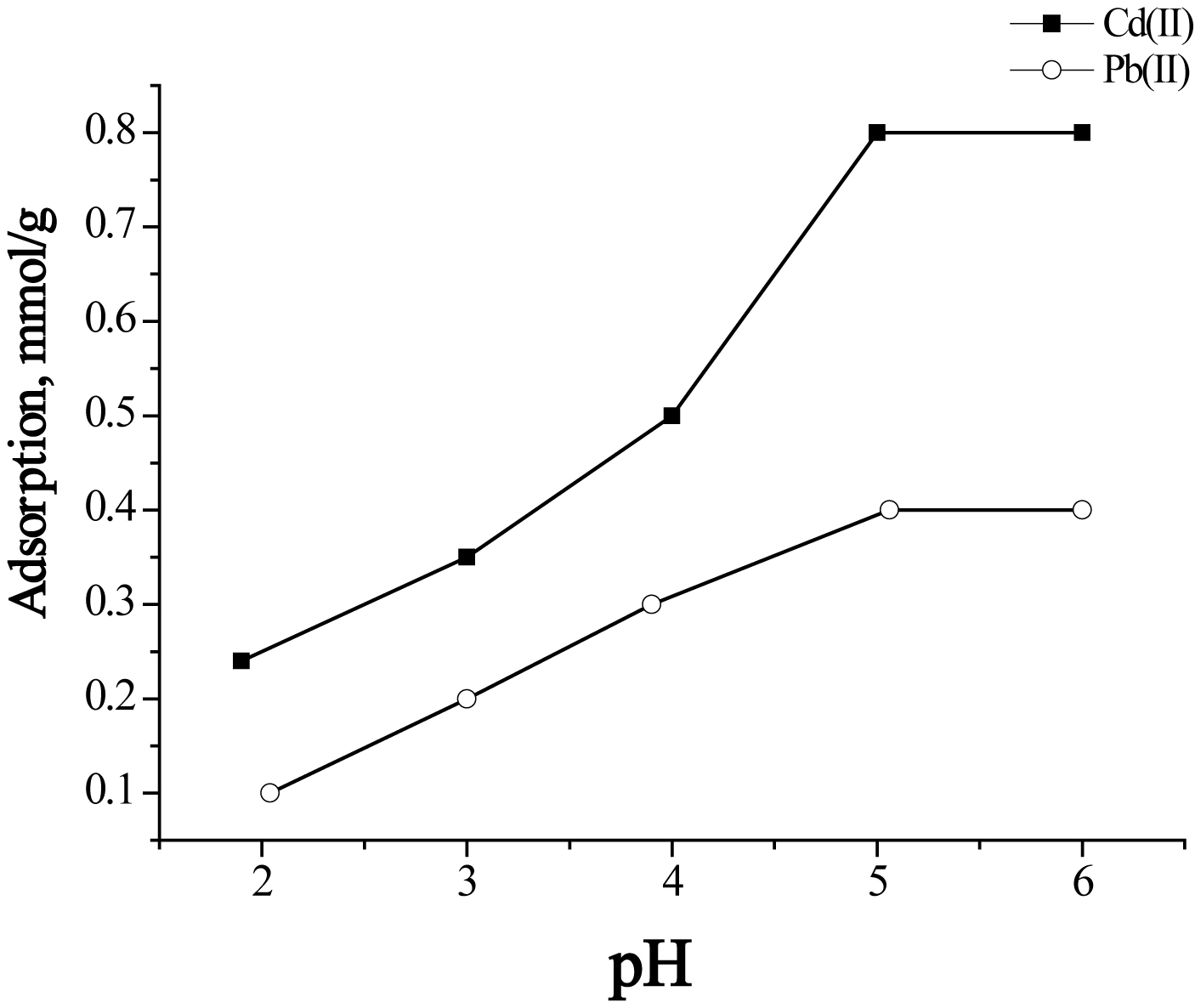


**Figure FS2.** Eﬀect of pH on adsorption of Cd(II) and Pb(II) ions by **SP1** sample.


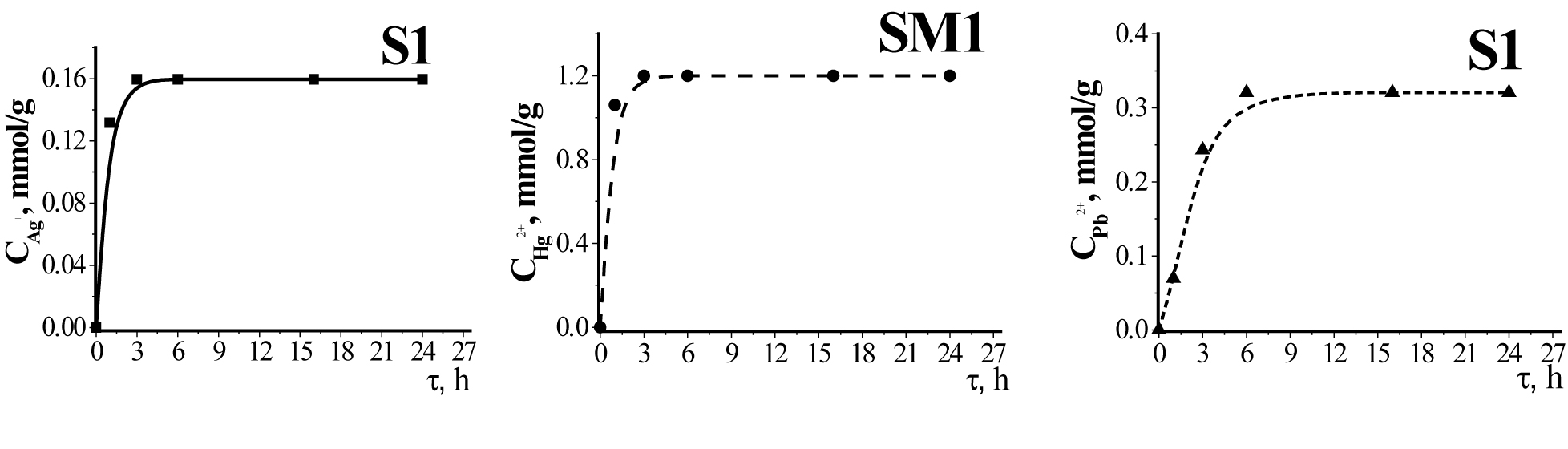


**Figure FS3.** Kinetic curves of Ag (I), Hg (II), and Pb (II) ions adsorption by magnetic adsorbents with thiol-containing groups in the surface layer
